# Development and Evaluation of a Duo SARS-CoV-2 RT-qPCR Assay Combining Two Assays Approved by the World Health Organization Targeting the Envelope and the RNA-Dependant RNA Polymerase (RdRp) Coding Regions

**DOI:** 10.3390/v12060686

**Published:** 2020-06-25

**Authors:** Laura Pezzi, Remi N. Charrel, Laetitia Ninove, Antoine Nougairede, Gregory Molle, Bruno Coutard, Guillaume Durand, Isabelle Leparc-Goffart, Xavier de Lamballerie, Laurence Thirion

**Affiliations:** 1 UR7310, Laboratoire de Virologie, Université de Corse-Inserm, 20250 Corte, France; laura.pezzi3@studio.unibo.it; 2 Unité des Virus Émergents (UVE: Aix-Marseille Univ-IRD 190-Inserm 1207-IHU Méditerranée Infection), 13005 Marseille, France; laetitia.ninove@ap-hm.fr (L.N.); antoine.nougairede@univ-amu.fr (A.N.); gregory.molle21@gmail.com (G.M.); bruno.coutard@univ-amu.fr (B.C.); guillaume.durand@inserm.fr (G.D.); isabelle.leparc-goffart@inserm.fr (I.L.-G.); xavier.de-lamballerie@univ-amu.fr (X.d.L.); laurence.thirion@ird.fr (L.T.); 3 Institut de Recherche Biomédicale des Armées, National Reference Laboratory for Arboviruses, 13005 Marseille, France

**Keywords:** coronavirus, COVID-19, real-time PCR, diagnostics, diagnosis, emerging, respiratory, molecular, outbreak, preparedness, response, TaqMan

## Abstract

The recent emergence of severe acute respiratory syndrome coronavirus 2 (SARS-CoV-2) worldwide has highlighted the importance of reliable and rapid diagnostic testing to prevent and control virus circulation. Dozens of monoplex in-house RT-qPCR assays are already available; however, the development of dual-target assays is suited to avoid false-negative results caused by polymorphisms or point mutations, that can compromise the accuracy of diagnostic and screening tests. In this study, two mono-target assays recommended by WHO (E-Sarbeco (enveloppe gene, Charite University, Berlin, Germany) and RdRp-IP4 (RdRp, Institut Pasteur, Paris, France)) were selected and combined in a unique robust test; the resulting duo SARS-CoV-2 RT-qPCR assay was compared to the two parental monoplex tests. The duo SARS-CoV-2 assay performed equally, or better, in terms of sensitivity, specificity, linearity and signal intensity. We demonstrated that combining two single systems into a dual-target assay (with or without an MS2-based internal control) did not impair performances, providing a potent tool adapted for routine molecular diagnosis in clinical microbiology laboratories.

## 1. Introduction

The recent spread of severe acute respiratory syndrome coronavirus 2 (SARS-CoV-2) worldwide exemplifies the need for early detection assays in order to prevent and control the circulation of pathogens [1]. In particular, real-time PCR assays allow a viral detection at the early stage of the infection, before the rise of antibodies; they are the most frequently used technique for routine diagnosis, because of their sensitivity, specificity and short-turnaround time. Since the beginning of the epidemic, several qualitative reverse-transcription polymerase chain reaction (RT-qPCR) assays have been developed [2,3,4]; the sensitivity and efficiency of most of them have been evaluated in a comparative study [5]. Polymorphisms, point mutations or major sequence deletions/insertions can compromise the accuracy of diagnostic and screening tests, resulting in false-negative NAT results, despite viral RNA at concentration levels sufficiently high for detection by NAT. To avoid this, we decided to develop a dual-target SARS-CoV-2 assay, combining two mono-target assays in the same reaction tube, as previously reported for chikungunya virus and Zaire ebolavirus [6,7]. Two monoplex assays already evaluated in literature and recommended by WHO were chosen to be included in the duo SARS-CoV-2 assay [2,4]; performances of these two parental assays and of the “designed in this study” duo RT-qPCR assay were compared.

## 2. Materials and Methods

### 2.1. Monoplex RT-qPCR Assays

The two monoplex RT-qPCR assays chosen to be included in the duo SARS-CoV-2 are (I) E-Sarbeco assay, developed by Charité Institute of Virology, Universitätsmedizin Berlin [3] and (II) RdRp-IP4 assay, developed by Institut Pasteur, Paris [2] (Table 1). RdRp-IP4 assay has been used here in monoplex format, while in the original protocol of the Institut Pasteur, it was multiplexed with RdRp-IP2 assay.

### 2.2. Generation of In Vitro Transcribed RNAs (IVT RNAs)

Two plasmids synthesized by Genscript were used; the first one containing the region of the E gene amplified by the E-Sarbeco assay, the second one containing the region of the RdRP gene amplified by the RdRp-IP4 assay (sequences available in the Supplementary Data). As described, the exogenic *NotI* hybridization sequence has been incorporated for the possible detection of laboratory carry-over contamination) [8]. From these plasmids, we in vitro transcribed RNA (IVT RNAs) to assess the sensitivity of RT-qPCR assays. The RNA transcript was synthesized in vitro by using the MEGAshortscript™ T7 Transcription Kit (Ref: AM1354, Invitrogen-Thermo Fisher Scientific, Carlsbad, CA, USA), according to the manufacturer’s instructions. TURBO DNase included in the same kit was used to remove any residual DNA. The RNA transcript was purified using MEGAclear™ Purification of Transcription reactions (Ref: AM1908, Invitrogen-Thermo Fisher Scientific, Carlsbad, CA, USA). The RNA concentration was determined using a Thermo Scientific™ NanoDrop™ (Thermo Fisher Scientific). The RNA transcript was serially diluted from 10^8^ to 10^2^ copies/µL, and dilutions were stored at −80 °C.

### 2.3. RT-qPCR

RT-qPCR was performed with SuperScript III Platinum One-Step RT-qPCR Kit with ROX (#11732-088, Invitrogen-Thermo Fisher Scientific), on a BioRad CFX96™ thermal cycler, software version 3.1 (Bio-Rad Laboratories). Primers were synthesized and provided by Eurogentec, probes by Life Technologies, ThermoFisher Scientific. For the duo SARS-CoV-2 assay, primers and probes were pooled together in the same reaction tube. A 25-µL reaction was set up containing 12.5 µL of 2× Reaction Mix, 0.5 µL of Superscript III RT/Platinum Taq Mix, primers and probe, at the concentrations described in Table 1 and 5 µL of RNA (IVT RNA E-Sarbeco pour E-Sarbeco assay, IVT RNA RdRp-IP4 pour RdRp-IP4 assay, pool of IVT RNA E-Sarbeco and IVT RNA RdRp-IP4 for the duo SARS-CoV-2 assay). The cycling conditions were: 50 °C for 15 min; 95 °C for 2 min; 45 cycles of 95 °C for 15 s and 58 °C for 45 s. All probes were labeled with the same dye (FAM). There are no modifications for either the sequence or the concentrations of the primers and probes. The only modification done concerns the quencher of the probes of both systems, in order to have the same quencher for the two assays included in the duo SARS-CoV-2. BBQ of the E-Sarbeco assay and BHQ-1 of the RdRp-IP4 assay were both replaced by QSY.

### 2.4. Analytical Sensitivity

The evaluation of the sensitivity of the two monoplex assays (E-Sarbeco and RdRp-IP4) was done using the IVT RNA E-Sarbeco and the IVT RNA RdRp-IP4. Serial dilutions of the quantified IVT RNAs were prepared using AE buffer (ref 740917.1, Macherey-Nagel™, Hoerdt, France). They contained 1.2 × 10^3^ to 1 copy/µL for IVT RNA RdRp-IP4 and 4.4 × 10^2^ to 1 copy/µL for IVT RNA E-Sarbeco. Seven decreasing concentrations were tested, using 12 replicates for each. A Ct ≥ 40 was considered as negative. The lower limit of detection was determined by probit regression analysis, using IBM SPSS Statistics software version 24. The LOD was defined as a concentration of viral copies, achieving a 95% hit rate (LOD95). To assess sensitivity of the duo SARS-CoV-2 assay, the 7 dilutions of IVT RNA RdRp-IP4 and the 7 dilutions of IVT RNA E-Sarbeco were pooled according to the dilution ratio (the most concentrated dilution of IVT RNA E-Sarbeco was pooled with the most concentrated dilution of IVT RNA RdRp-IP4 and so on, until the highest dilution of IVT RNA E-Sarbeco was pooled with the highest dilution of IVT RNA RdRp-IP4).

### 2.5. Clinical Samples for SARS-CoV-2 RNA Detection

A total of 16 nasopharyngeal samples were collected in patients recently presenting, or having presented, clinical signs compatible with COVID-19. They were initially tested for SARS-CoV-2 RNA using the E gene RT-qPCR. All patients were also sampled for serology. Both types of samples were used secondarily for validation of assays under development, such as those described in this study.

Another 53 nasopharyngeal samples were collected from asymptomatic individuals, and tested using E gene and duo SARS-CoV-2 assays. Sera from these individuals were also tested via a seroneutralization test.

### 2.6. Neutralization Assay

The virus neutralization assay was adapted from the protocol described previously [9] and performed in a BSL3 facility. The assay was performed in 96-well microtiter plates using Vero cells (ATCC CCL-81). Briefly, sera were two-fold diluted from 1:10 to 1:80 and were then mixed with an equal volume of 50 TCID_50_ (tissue culture infective dose producing pathological change in 50% of the cell culture inoculated) of virus (BavPat1/2020 strain from EVAg, Ref-SKU: 022N-03902, https://www.european-virus-archive.com/), and incubated for one hour at 37 °C, and inoculated onto 1.3 × 10^5^ cells/well. The controls consisted of 1:10 serum/Vero cells/no virus and titration of the diluted virus. After 4 days of incubation at 37 °C with 5% CO_2_, the microplates were read, and the presence (neutralization titer at 20, 40, 80, and 160) or absence (no neutralization) of the cytopathic effect was noted. The cutoff value for positivity was set at titer 40, as previously described using the same technique [10,11].

## 3. Results

### 3.1. Analytical Sensitivity

**Figure 1:**
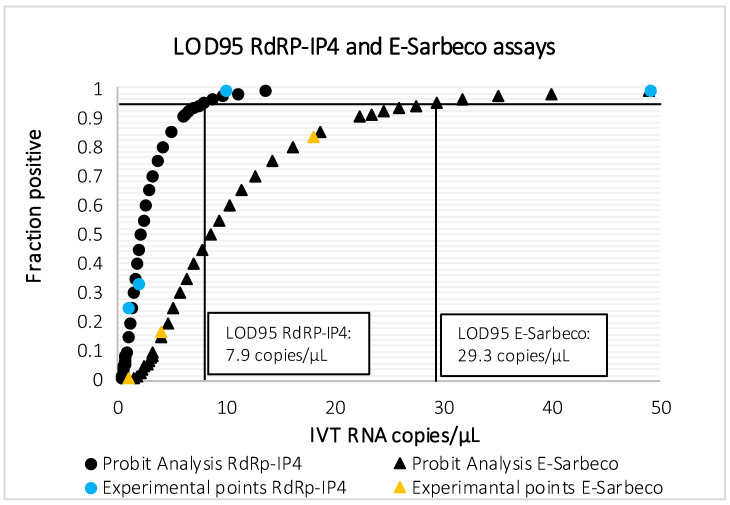
Probit analysis for LOD95 of RdRp-IP4 and E-Sarbeco assays.

The results are presented in Table 2 and Figure 1. LOD95 of E-Sarbeco assay was 29.3 RNA copies/µL [16.5–139], although it could detect 2/12 replicates containing 4 IVT RNA copies/µL. RdRp-IP4 assay proved to be more sensitive, with a LOD95 of 7.9 RNA copies/µL [4.2–84.4], also detecting 3/12 replicates containing 1 IVT RNA copy/µL. Sensitivity of the duo SARS-CoV-2 was comparable to that of RdRp-IP4 assay (1 copy/µL of IVT RNA RdRp-IP4).

### 3.2. Linearity and Signal Intensity

**Figure 2:**
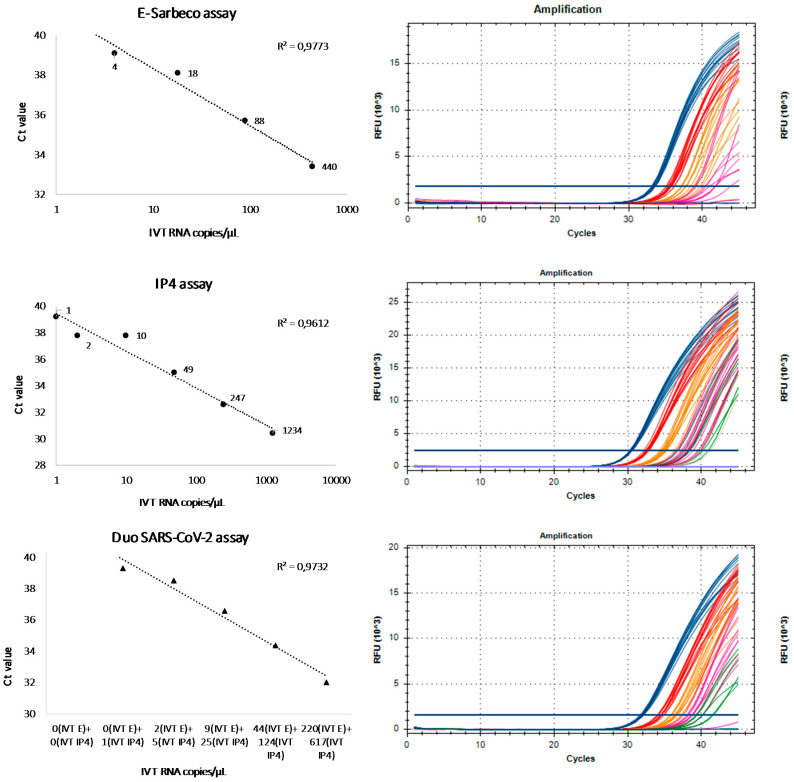
Linearity and signal intensity of E-Sarbeco, RdRp-IP4 and duo SARS-CoV-2 assays.

**Figure 3:**
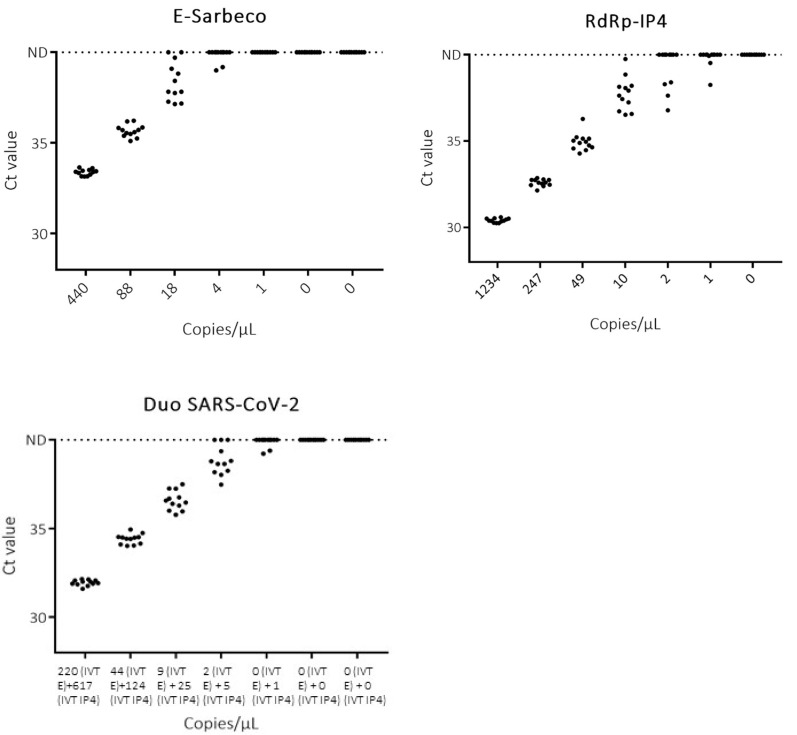
Detection of dilutions of IVT RNA E-Sarbeco and RdRp-IP4 with E-Sarbeco, RdRP-IP4 and duo SARS-CoV-2 assays. ND: not detected.

The results about the analytical sensitivity test obtained with the duo SARS CoV-2 were compared to the results of the monoplex assays, E-Sarbeco and RdRp-IP4, in order to evaluate the correlation between Ct values and the seven different RNA IVT concentrations previously tested. The response (Ct) for the three systems is linear, with the correlation coefficient (*R*^2^) close to one (Figure 2 and Figure 3). Moreover, as shown in Figure 2, the three assays also provide a strong signal intensity for low copy dilutions close to the limit of detection. It is important to discriminate between negative and positive results without ambiguity.

### 3.3. Clinical Samples

Among 16 clinical samples tested, 10 were positive when tested with the E-Sarbeco monoplex assay. duo SARS-CoV-2 assay could detect the same 10 samples, and 4 additional samples with Ct in the range 36.8–38.9. Two of these four samples were also detected by a RdRp-IP4 assay, so were classified as positive by RT-qPCR. The other two samples were negative with the RdRp-IP4 assay, but SARS-CoV-2 infection was further confirmed by the seroneutralization assay. Among the two samples testing negative with all the RT-qPCR assays, one was also negative in the seroneutralization assay, while the second one was positive. All 16 specimens, including the negative ones, have been validated, i.e., the IC, expected to range from 29–31 Ct values, were within the 28–32 Ct values range; this demonstrate that the extraction and the RT-qPCR reactions performed well and that there was no significant amount of inhibitors in the tested sample; specifically, the #3,4,7,8,15,16 had IC Ct values that ranged from 29.3 to 30.4. The detailed results of molecular testing on clinical samples are presented in Table 3 and in the Supplementary Data. The 53 samples collected from asymptomatic individuals, with no evidence of infection via a seroneutralization test, tested negative with both the E gene and duo SARS-CoV-2 assay.

## 4. Discussion

Duo assays have been developed for COVID-19. but to the best of our knowledge, all duo assays are only commercially available (Cepheid GeneXpert, Panther fusion, Roche cobas®, Abbott etc.); here, we provide data supporting the fact that duo assays can be developed by combining two in-house assays. Our study provides leads for those interested in such development, and is intended as a model for validation. We developed and evaluated a duo RT-qPCR assay for the detection of SARS-CoV-2, by combining two monoplex assays, targeting the envelope and the RdRp genes. The advantages of targeting more than one region of the genome for a diagnostic purpose have been extensively described in previous studies [6,7,12,13]. Briefly, the main advantage is to prevent false negative results: mutations often observed with emerging pathogens, especially with RNA viruses, can lead to possible mispriming and a lack of viral detection. The inclusion of two targets into the assay design assures that the failure of one region detection is compensated by the detection of the other. Secondly, the use of a unique fluorescent dye for the two probes (in this case, FAM) makes the interpretation of the results easier. A negative RT-qPCR result corresponds to the lack of amplification by both assays; a positive result corresponds to the detection of a viral genome by either both assays or one assay only. Thirdly, as observed in our study, the sensitivity and intensity of fluorescent signals are not impaired in the duo assay.

Using a dual-target assay can help prevent false-negative results due to polymorphism, point mutations, or major sequence deletions/insertions. This is not specific of SARS-CoV-2 and not a peculiar feature of the two assays selected in this study. This is a theoretical advantage given the fact that RNA viruses are prone to genetic variability. The six genomic regions corresponding to primers and probes were studied using either (i) the 14,277 COG-UK Consortium sequences, (ii) or 73 complete genome sequences [14]; very little genetic heterogeneity was observed (Supplementary Dataset #2).

The WHO currently lists 14 RT-qPCR assays to diagnose COVID-19 [2]. Of these, nine assays were compared for their limit of detection using RNA transcript standards [5]; the best performances were observed for E-Sarbeco (Charité), HKU-ORF1/nsp14 (HKU) and 2019-nCoV_N1 (US CDC) [5]. In the meantime, the Pasteur Institute designed two assays targeting the RdRp, named IP2 and IP4; it was recommended that these assays be used for screening, and the E-Sarbeco assay for the confirmation of undetermined results [2]. RdRp-IP4 proved to be more sensitive than RdRp-IP2 in our laboratory internal validation system (data not shown); so, RdRp-IP4 was selected to be combined with E-Sarbeco. The excellent sensitivity of both E-Sarbeco and RdRp-IP4, with LOD95 at 23.9 and 7.9 RNA copies/µL, respectively, is reported in our study. These results are in line with results published by other groups [5]. At the outset of our study, there were no peer-reviewed data for the analytical performances of RdRp-IP4, although it is used extensively in France and beyond, with excellent reports from users (personal data). It has been tested in multiplex format with the RdRp-IP2 assay, claiming a LOD95 of 100 copies of RNA genome equivalent/reaction [2]. The LOD95 we obtained for RdRp-IP4 monoplex assay (7.9 IVT RNA copies/µL, Figure 1) encouraged us to include this assay in our duo SARS-CoV-2, together with the E-Sarbeco assay. At the outset of this study, the two IVT RNA (E-Sarbeco and RdRp-IP4) were available as positive control for each assay independently. When the decision to set-up a duo assay was taken, we combined the two independent IVT RNA: thus, the concentration was not perfectly adapted (Table 2, Figure 2). Designing, de novo, a single IVT RNA encompassing the two selected target sequences would allow equimolar concentrations and avoid tedious calculation to attempt for equimolarity. However, we wanted to show that the validation of a duo assay can be performed rapidly within an emergency situation, by combining two assays developed separately together with their respective corresponding IVT RNA as positive controls. Finally, ECDC, in its most recent update on the COVID-19 bulletin, backed down on the WHO recommendation concerning the need to test at least 2 genomic targets separately [15]. This decision was taken because of the reagent shortage increasingly reported in European laboratories. Regarding this problem, the advantage of the duo assay is the ability to test simultaneously two targets plus the internal control in one single reaction (see below), with the amount of reagent and consumable necessary being for one assay only.

Since the specificity of RdRp-IP4 (influenza A(H1N1) pdm09, A(H3N2), B-Victoria, B-Yamagata; influenza C; RSV-A, RSV-B; hBoV; hPIV; hMPV; HRV/enterovirus; adenovirus; hCoV (MERS-CoV, HKU1, OC43, 229E and NL63)) and E-Sarbeco had been evaluated previously [2,3], we did not repeat the study, considering that the combination of two assay would have no deleterious impact on the specificity of the resulting duo assay, as previously shown [6,7].

Although the number of clinical samples included in our study is limited (*n* = 69), it shows that the duo SARS-CoV-2 assay has a better LoD compared to E-Sarbeco and RdRp-IP4 monoplex assays. All the samples testing positive with E-Sarbeco and RdRp-IP4 assays were also detected by the duo SARS-CoV-2, that could also identify 4 additional weakly positive samples, that otherwise would have been considered negative based on E-Sarbeco results (#7, #8, #15, #16, Table 3). RdRp-IP4, even if more sensitive than E-Sarbeco assay, could detect only 2/4 of these weakly positive samples (#15 and #16, Table 3), whereas they were undoubtedly positive with the duo SARS-CoV-2. Low viral loads can either represent the very early stage of a new infection or the last phase of the disease, when symptoms have disappeared, but the virus is still present [16,17]. In Table 3, the sixteen samples were collected in a small battalion of firemen that had endured COVID-19 cases; these samples correspond to the second series of collection in these young soldiers. Accordingly, these samples were collected late after the clinical onset, 7 to 17 days (Table 3). Since early samples exhibit high viral loads and low Ct values, they are usually not very challenging for the direct diagnosis of SARS-CoV-2 infection [16]; in contrast, late samples are more challenging for diagnosis. This is the reason why we chose such clinical samples. It must be underlined that these 16 samples are by no means representative of the samples received in the laboratory for routine testing; however, they might be representative of samples for which RT-qPCR tests are more difficult to interpret. The use of more sensitive assays allows to early identify potential new cases, and to follow-up convalescent patients until complete viral clearance. Of the fifty-five samples tested negative using the three RT-qPCR assays, the lack of detectable neutralizing antibodies has confirmed their negative status vis-à-vis SARS-CoV-2 infection. In patient#4 (negative result with the three RT-qPCR assays, while positive by seroneutralization), the nasopharyngeal specimen was collected 13 days after clinical onset, accounting for the absence of detectable RNA.

A triplex format including the duo SARS-CoV-2 assay developed in this study (2 FAM-labeled probes) and an MS2 internal control of extraction (VIC-labeled probe) was also tested (data not shown) [8]. The addition of a third primers and probe set to detect MS2 bacteriophage caused a slight decrease in the fluorescent signal, but no differences were observed for Ct values between duo SARS-CoV-2 assay alone and duo assay coupled with an internal control. These preliminary results suggest that clinical samples can be tested using a unique RT-qPCR reaction; this might be of utmost importance to overcome the problems due to molecular biology reagent shortage, and to allow larger testing capacity for clinical microbiology laboratories involved in the COVID-19 response actions. The primers and probes presented in this study are publicly available for academics and industrials in the European Virus Archive catalog in the lyophilized format previously described [18] and can beneficiate from the free of charge access through the Trans National Access policy of the EVA-GLOBAL project [19,20,21].

Our study holds some limitations, such as (i) the lack of specificity evaluation for the duo SARS-CoV-2, (ii) an evaluation conducted with a small number of samples collected from the same country, thus accounting for limited variability. All experiments were done using the SuperScript III Platinum One-Step RT-qPCR kit (Thermo Fischer Scientific). If used with a different kit, the duo SARS-CoV-2 assay should be validated, since results are likely to vary according to the protocol.

Other RT-qPCR assays for detecting SARS-CoV-2 RNA can be considered to mount alternative duo assays. As previously reported, the primary objective is to develop an assay that is at least as sensitive as the two parental assays used separately. Accordingly, it would appear interesting to develop other duo assay by combining the monoplex RT-qPCR assays with the optimal performances. So far, candidate assays can be selected within the WHO recommended assays, providing the best performances in evaluation studies.

To conclude, combining two monoplex systems in a duo SARS-CoV-2 RT-qPCR provides a simple and robust molecular detection assay, with performances that are at least equal to, and most likely better than, each of the parental assays. The Ct values were not lower compared with the monoplex assays, but the analytical sensitivity was at least equal with IVT RNA controls (Table 2), and appeared to be better with clinical samples (Table 3). Interestingly, to the best of our knowledge, this study is the first to provide the analytical sensitivity of the RdRp-IP4 assay. As previously described for other viruses [6,7], this approach is well adapted for routine molecular diagnosis in clinical microbiology laboratories.

## Figures and Tables

**Table 1:** Primers and probe included in the duo severe acute respiratory syndrome coronavirus 2 (SARS-CoV-2).

Reference	Institute	Primer/Probe	5′→3′ Sequence	Target	Position ^a^	Amplicon Size	Concentration
[3]	Charité (Berlin)	E_Sarbeco_F	ACAGGTACGTTAATAGTTAATAGCGT	E gene	26,269–26,294	113 nt	400 nM
		E_Sarbeco_R	ATATTGCAGCAGTACGCACACA		26,360–26,381		400 nM
		E_Sarbeco_P	FAM-ACACTAGCCATCCTTACTGCGCTTCG-QSY		26,332–26,357		200 nM
[2]	Pasteur (Paris)	nCoV_IP4-14059Fw	GGTAACTGGTATGATTTCG	RdRp gene	14,080–14,098	107 nt	400 nM
		nCoV_IP4-14146Rv	CTGGTCAAGGTTAATATAGG		14,167–14,186		400 nM
		nCoV_IP4-14084Probe	FAM-TCATACAAACCACGCCAGG-QSY		14,105–14,123		200 nM

^a^ According to the sequence of SARS-CoV-2 Wuhan-Hu-1 (GenBank accession number NC_045512.2).

**Table 2:** Analytical sensitivity of E-Sarbeco (Charité), RdRp-IP4 (Institut Pasteur) and duo SARS-CoV-2 assays. In bold, lowest RNA copy number providing 12/12 positive results; in italicized bold, lowest RNA copy number providing at least 1/12 positive result.

	**E-Sarbeco Assay (Charité)**			
IVT RNA copies/µL	Total samples tested, No.	Positive samples, No.	% Detected	Ct, Mean (SD)
440	12	12	100	33.4 (0.2)
**88**	**12**	**12**	**100**	**35.7 (0.3)**
18	12	10	83	38.1 (1.0)
***4***	***12***	***2***	***17***	***39.1 (0.1)***
1	12	0	0	-
0	12	0	0	-
0	12	0	0	-
	**RdRp-IP4 Assay (Institut Pasteur)**			
IVT RNA copies/µL	Total samples tested, No.	Positive samples, No.	% Detected	Ct, Mean (SD)
1234	12	12	100	30.4 (0.1)
247	12	12	100	32.6 (0.2)
49	12	12	100	35 (0.5)
**10**	**12**	**12**	**100**	**37.8 (1)**
2	12	4	33	37.8 (0.7)
***1***	***12***	***3***	***25***	***39.2 (0.9)***
0	12	0	0	-
	**Duo SARS-CoV-2 Assay (This Study)**			
IVT RNA copies/µL	Total samples tested, No.	Positive samples, No.	% Detected	Ct, Mean (SD)
220 (IVT RNA E-Sarbeco) + 617 (IVT RNA RdRp-IP4)	12	12	100	32 (0.2)
44 (IVT RNA E-Sarbeco) + 124 (IVT RNA RdRp-IP4)	12	12	100	34.4 (0.3)
**9 (IVT RNA E-Sarbeco) + 25 (IVT RNA RdRp-IP4)**	**12**	**12**	**100**	**36.6 (0.6)**
2 (IVT RNA E-Sarbeco) + 5 (IVT RNA RdRp-IP4)	12	9	75	38.5 (0.6)
***0 (IVT RNA E-Sarbeco) + 1 (IVT RNA RdRp-IP4)***	***12***	***2***	***17***	***39.3 (0.1)***
0 (IVT RNA E-Sarbeco) + 0 (IVT RNA RdRp-IP4)	12	0	0	-
0 (IVT RNA E-Sarbeco) + 0 (IVT RNA RdRp-IP4)	12	0	0	-

**Table 3:** RT-qPCR results observed on clinical samples collected from symptomatic individuals, tested with E-Sarbeco, RdRp-IP4 and duo SARS-CoV-2 assays. Highlighted in grey, samples with discrepant RT-qPCR results.

Sample ID	Onset to Sample Collection (Days)	E-Sarbeco Assay (Ct Value)		RdRp-IP4 Assay (Ct Value)		Duo SARS-CoV-2 Assay (Ct Value)		VNT Titre
#1	16	Positive	35.2	Positive	34.9	Positive	35.2	80
#2	9	Positive	31.4	Positive	31.2	Positive	31.2	>160
#3	11	Negative	>40	Negative	>40	Negative	>40	<20
#4	13	Negative	>40	Negative	>40	Negative	>40	80
#5	13	Positive	31	Positive	30.4	Positive	30.8	80
#6	9	Positive	31	Positive	30.8	Positive	31	80
#7	17	Negative	>40	Negative	>40	Positive	38.1	>160
#8	15	Negative	>40	Negative	>40	Positive	37.2	>160
#9	12	Positive	35.4	Positive	37.4	Positive	35.8	>160
#10	16	Positive	32.8	Positive	34.2	Positive	33.1	40
#11	13	Positive	34	Positive	35.8	Positive	34.4	40
#12	15	Positive	37.1	Positive	36.4	Positive	35.9	40
#13	13	Positive	21.5	Positive	22.5	Positive	21.9	>160
#14	14	Positive	36.2	Positive	34.7	Positive	34.8	<20
#15	7	Negative	>40	Positive	35.2	Positive	36.8	>160
#16	9	Negative	>40	Positive	36.6	Positive	38.9	80

## Supplementary Materials

The following are available online at https://www.mdpi.com/1999-4915/12/6/686/s1, Doc S1 Sequences of the two IVT RNA used in the study.
