# Supplementary material for: Development and Evaluation of a Duo SARS-CoV-2 RT-qPCR Assay Combining Two Assays Approved by the World Health Organization Targeting the Envelope and the RNA-Dependant RNA Polymerase (RdRp) Coding Regions
Source: Viruses. 2020 Jun 25;12(6):686. doi: 10.3390/v12060686 (PMC7354606; doi:10.3390/v12060686)
Supplement: Supplementary file 1 [file viruses-12-00686-s001.pdf]

Title: Development and evaluation of a Duo SARS-CoV-2 RT-qPCR assay combining two assays approved by the World Health Organization targeting the envelope and the RNA-dependant RNA polymerase (RdRp) coding regions

Authors: Laura Pezzi, Remi N. Charrel, Laetitia Ninove, Antoine Nougairede, Gregory Molle, Bruno Coutard, Guillaume Durand, Isabelle Leparc-Goffart, Xavier de Lamballerie, Laurence Thirion

Supplementary data

Suppl. Dataset#1. Sequences of the two IVT RNA used in the study.

Highlighted in blue: primers
Highlighted in green: probe
Bold underlined, T7 promoter sequence
Italicized, T7 termination sequence

>IVT_E gene
TAATACGACTCACTATAGGGAGAACATGAGGATCACCCATGTACAGGTACGTTAATAGTTAATAGCGTACTTCTTTTTCTTATTATAGCGGCCGCTTATTAAGTTACACTA
GCCATCCTTACTGCGCTTCGATTGTGTGCGTACTGCTGCAATATACATGAGGATCACCCATGT*gcaataactagcataaccccttggggcctctaaacgggtcttgaggggttttttgctga*

>IVT_RdRp-IP4
TAATACGACTCACTATAGGGAGAACATGAGGATCACCCATGTCAAGTATTGA**ATTATAGCGGCCGCTTATTA**CGAAATGCTGGTATTGTTGGTGTACTGACATTAG
ATAATCAAGATCTCAATGGTAACTGGTATGATTTCGGTGATTTCATACAAACCACGCCAGGTAGTGGAGTTCCTGTTGTAGATTCTTATTATTCATTGTTAATGCC
TATATTAACCTTGACCAGGGCTTACATGAGGATCACCCATGT*gcaataactagcataaccccttggggcctctaaacgggtcttgaggggttttttgctga*

Suppl. Figure 1. Flowchart of molecular testing on clinical samples using E-Sarbeco, RdRp-IP4 and Duo SARS-CoV-2 assays.

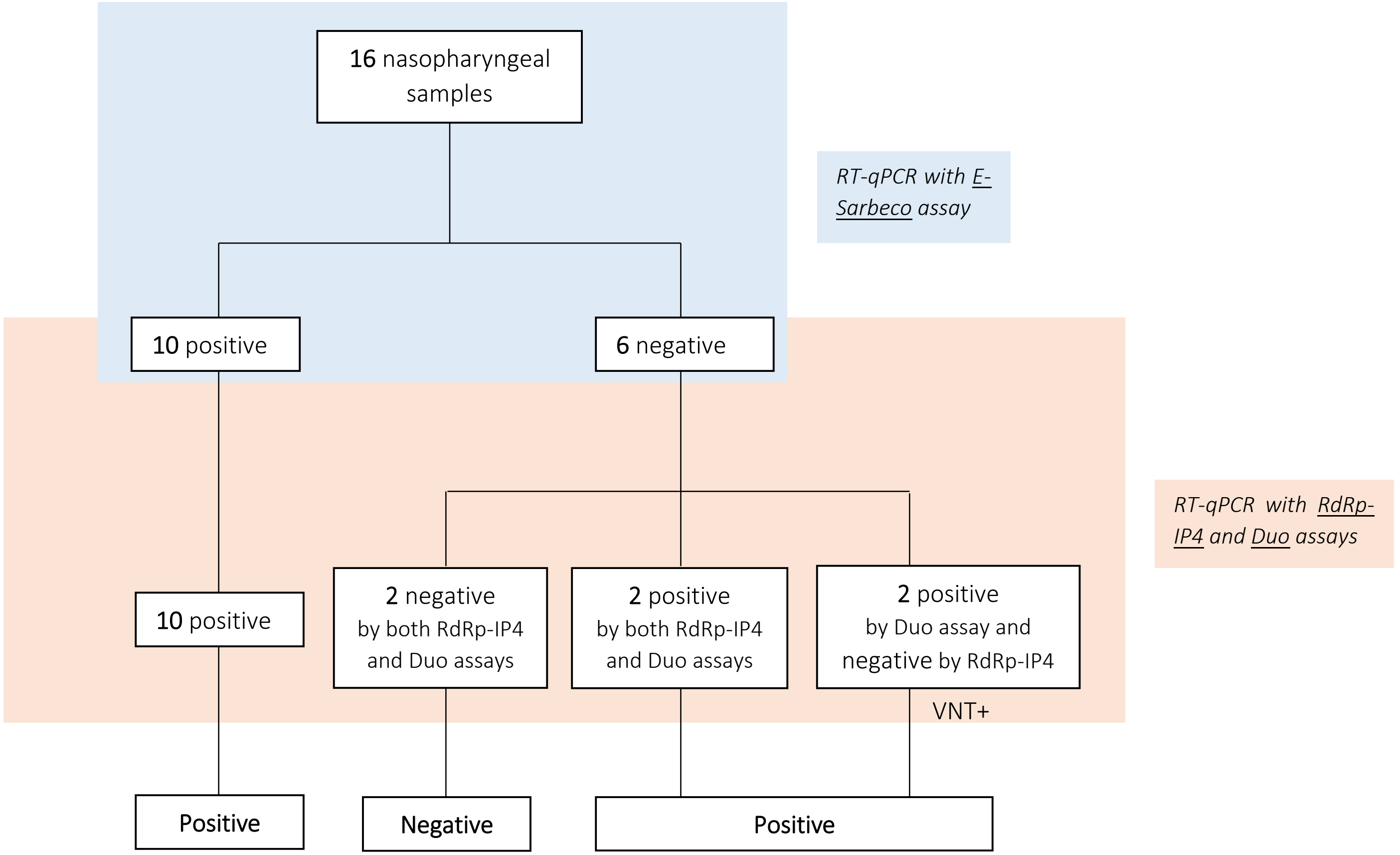

Table 1. Results of RT-qPCR on clinical samples with E-Sarbeco, RdRp-IP4 and Duo SARS-CoV-2 assays. Highlighted in grey, samples with discrepant results obtained with the three assays.

Sample nb	Sample name	Days between onset of symptoms and sampling (sera and nasopharyngeal swabs)	E-Sarbeco assay		RdRp-IP4 assay		Duo SARS-CoV-2		VNT	Titre VNT
1	COVID-0002	16	Positive	35,2	Positive	34,9	Positive	35,2	Positive	80
2	COVID-0004	9	Positive	31,4	Positive	31,2	Positive	31,2	Positive	>160
3	COVID-0006	11	Negative	>40	Negative	>40	Negative	>40	Negative	-
4	COVID-0008	13	Negative	>40	Negative	>40	Negative	>40	Positive	80
5	COVID-0010	13	Positive	31	Positive	30,4	Positive	30,8	Positive	80
6	COVID-0012	9	Positive	31	Positive	30,8	Positive	31	Positive	80
7	COVID-0016	17	Negative	>40	Negative	>40	Positive	38,1	Positive	>160
8	COVID-0018	15	Negative	>40	Negative	>40	Positive	37,2	Positive	>160
9	COVID-0020	12	Positive	35,4	Positive	37,4	Positive	35,8	Positive	>160
10	COVID-0022	16	Positive	32,8	Positive	34,2	Positive	33,1	Positive	40
11	COVID-0024	13	Positive	34	Positive	35,8	Positive	34,4	Positive	40
12	COVID-0026	15	Positive	37,1	Positive	36,4	Positive	35,9	Positive	40
13	COVID-0029	13	Positive	21,5	Positive	22,5	Positive	21,9	Positive	>160
14	COVID-0031	14	Positive	36,2	Positive	34,7	Positive	34,8	Negative	-
15	COVID-0033	7	Negative	>40	Positive	35,2	Positive	36,8	Positive	>160
16	COVID-0035	9	Negative	>40	Positive	36,6	Positive	38,9	Positive	80

Suppl Figure 2. Detection curves of samples with discrepant results obtained with the three RT-qPCR assays.

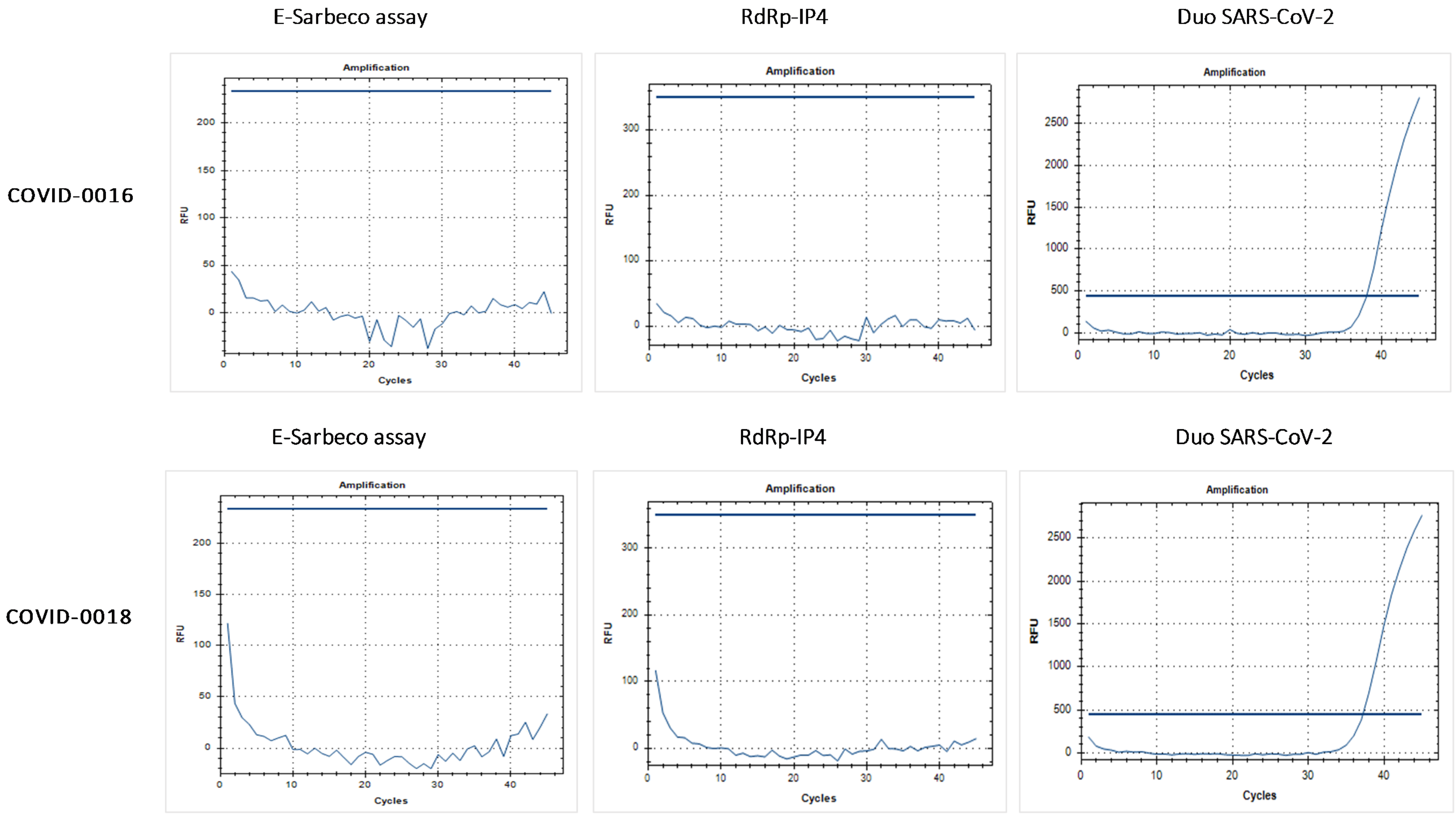

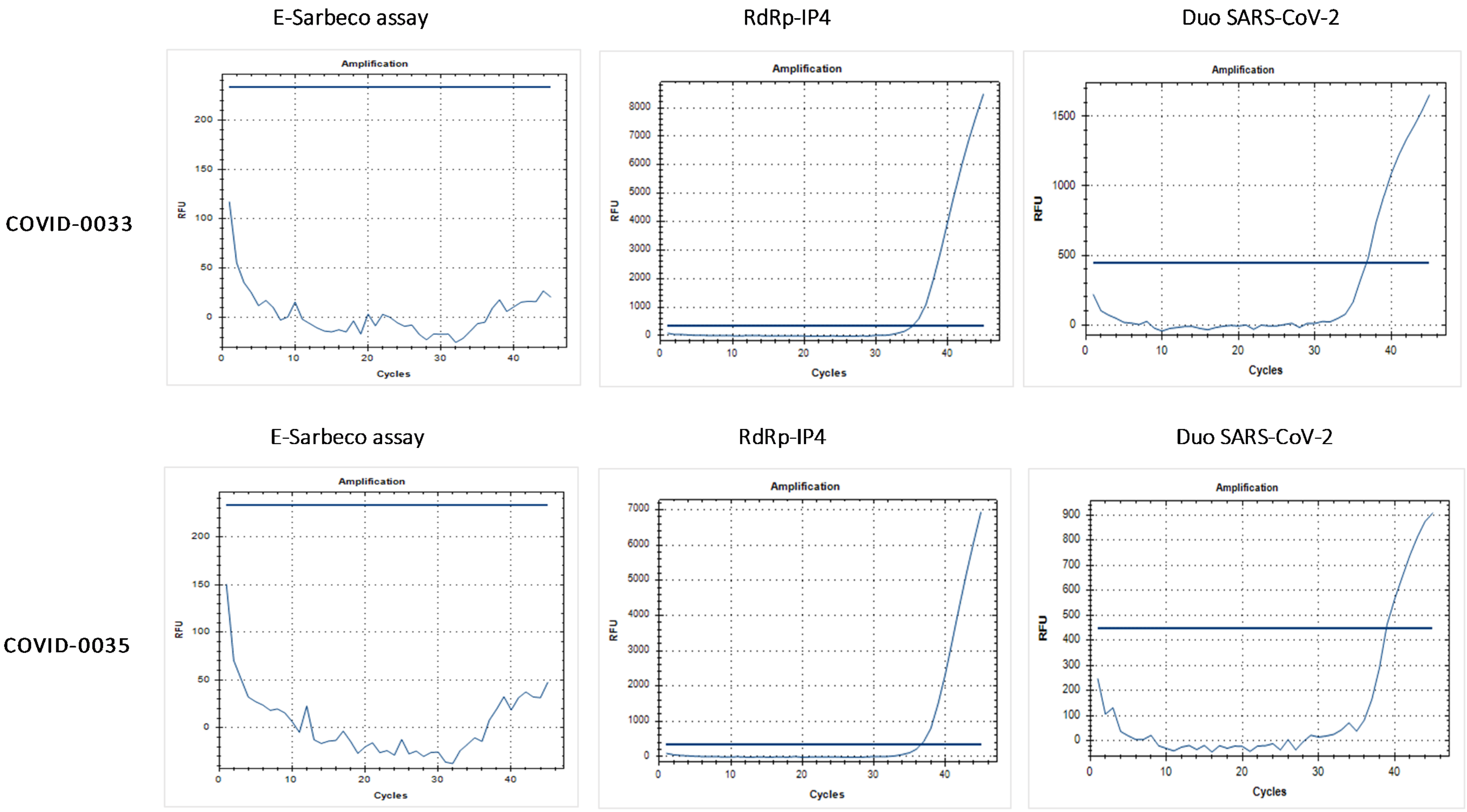

E-Sarbeco assay
RdRp-IP4
Duo SARS-CoV-2
COVID-0033
COVID-0035
Amplification
RFU
Cycles

Suppl. Dataset#2. *In silico* analysis of the genetic heterogeneity in the target regions of the assay included in the Duo SARS-CoV-2 assay

Analysis #A: Based on 14,277 complete genome sequences of SARS-CoV-2 from COG-UK Consortium (Regions: Scotland, England, Wales, Northern Ireland)

A1. IP4-RdRp

99,16% of sequences (14,156 sequences): no mismatch with IP4-RdRp primers and probe set

0,84% of sequences (121 sequences): 1 or more mismatches with IP4-RdRp primers and probe set

- 8 sequences: 1 or more mismatches with Fw primer
- 37 sequences: 1 or more mismatches with probe
- 76 sequences: 1 or more mismatches with Rev primer

A2. E gene (Charité, Berlin)

99,83% of sequences (14,252 sequences): no mismatch with E gene(Charité) primers and probe set

0,17% of sequences (25 sequences): 1 or more mismatches with E gene(Charité) primers and probe set

- 4 sequences: 1 or more mismatches with Fw primer
- 19 sequences: 1 or more mismatches with probe
- 2 sequences: 1 or more mismatches with Rev primer

Analysis #B: Based on 14,277 complete genome sequences of SARS-CoV-2 from COG-UK Consortium (Regions: Scotland, England, Wales, Northern Ireland)

B1. E-Sarbeco assay regions corresponding to the primers and the probe (73 sequences)

	27,859 … 27,884		27,922 … 27,947		27,950 … 27,971
E Sarbeco (Charité)	A C A G G T A C G T T A A T A G T T A A T A G C G T	//	A C A C T A G C C A T C C T T A C T G C G C T T C G	//	T G T G T G C G T A C T G C T G C A A T A T
NC_045512_|Severe_acute_respiratory_syndrome_coronavirus_2_isolate_Wuhan-Hu-1|_complete_genome	....		....		....
LC529905_|Severe_acute_respiratory_syndrome_coronavirus_2_TKYE6182_2020_RNA|_complete_genome	....		....		....
MT371050_|Severe_acute_respiratory_syndrome_coronavirus_2_isolate_SARS-CoV-2/human/LKA/COV486/2020|_complete_genome	....		....		....
LC542809_|Severe_acute_respiratory_syndrome_coronavirus_2_TKYE6947_2020_RNA|_complete_genome	....		....		....
MT135042_|Severe_acute_respiratory_syndrome_coronavirus_2_isolate_SARS-CoV-2/human/CHN/231/2020|_complete_genome	....		....		....
MT350282_|Severe_acute_respiratory_syndrome_coronavirus_2_isolate_SARS-CoV-2/human/BRA/SP02cc/2020|_complete_genome	....		....		....
MT114412_|Severe_acute_respiratory_syndrome_coronavirus_2_isolate_SARS-CoV-2/human/HKG/HKU-902a/2020|_complete_genome	....		....		....
MT192772_|Severe_acute_respiratory_syndrome_coronavirus_2_isolate_SARS-CoV-2/human/VNM/nCoV-19-01S/2020|_complete_genome	....		....		....
MT192773_|Severe_acute_respiratory_syndrome_coronavirus_2_isolate_SARS-CoV-2/human/VNM/nCoV-19-02S/2020|_complete_genome	....		....		....
MT320538_|Severe_acute_respiratory_syndrome_coronavirus_2_isolate_SARS-CoV-2/human/FRA/KRA-ROB/2020|_complete_genome	....		....		....
MT276330_|Severe_acute_respiratory_syndrome_coronavirus_2_isolate_SARS-CoV-2/human/USA/FL_5091/2020|_complete_genome	....		....		....
MT304491_|Severe_acute_respiratory_syndrome_coronavirus_2_isolate_SARS-CoV-2/human/USA/TX_2967/2020|_complete_genome	....		....		....
MT159722_|Severe_acute_respiratory_syndrome_coronavirus_2_isolate_2019-nCoV/USA-CruiseA-6/2020|_complete_genome	....		....		....
MT452574_|Severe_acute_respiratory_syndrome_coronavirus_2_isolate_SAR-CoV-2/human/USA/NY-QDX-00000001/2020|_complete_genome	....		....		....
MT422807_|Severe_acute_respiratory_syndrome_coronavirus_2_isolate_SARS-CoV-2/human/USA/UF-5/2020|_complete_genome	....		....		....
MT339041_|Severe_acute_respiratory_syndrome_coronavirus_2_isolate_SARS-CoV-2/human/USA/AZ-ASU2936/2020|_complete_genome	....		....		....
MT328035_|Severe_acute_respiratory_syndrome_coronavirus_2_isolate_SARS-CoV-2/human/GRC/13/2020|_complete_genome	....		....		....
MT121215_|Severe_acute_respiratory_syndrome_coronavirus_2_isolate_SARS-CoV-2/human/CHN/SH01/2020|_complete_genome	....		....		....
MT039874_|Severe_acute_respiratory_syndrome_coronavirus_2_isolate_SARS-CoV-2/human/CHN/IME-HZ01/2020|_complete_genome	....		....		....
MT066175_|Severe_acute_respiratory_syndrome_coronavirus_2_isolate_SARS-CoV-2/NTU01/TWN/human/2020|_complete_genome	....		....		....
MT020781_|Severe_acute_respiratory_syndrome_coronavirus_2_isolate_nCoV-FIN-29-Jan-2020|_partial_genome	....		....		....
MT066156_|Severe_acute_respiratory_syndrome_coronavirus_2_isolate_SARS-CoV-2/human/ITA/INMI1/2020|_complete_genome	....		....		....
MT281577_|Severe_acute_respiratory_syndrome_coronavirus_2_isolate_SARS-CoV-2/human/CHN/Fuyang_FY002/2020|_complete_genome	....		....		....
MT304474_|Severe_acute_respiratory_syndrome_coronavirus_2_isolate_SARS-CoV-2/human/KOR/BA-ACH_2604/2020|_complete_genome	....		....		....
MT396241_|Severe_acute_respiratory_syndrome_coronavirus_2_isolate_SARS-CoV-2/human/CHN/YN-0306-466/2020|_complete_genome	....		....		....
MT434782_|Severe_acute_respiratory_syndrome_coronavirus_2_isolate_SARS-CoV-2/human/USA/NY-CDC-SURV0076NYC/2020|_complete_genome	....		....		....
MT106054_|Severe_acute_respiratory_syndrome_coronavirus_2_isolate_2019-nCoV/USA-TX1/2020|_complete_genome	....		....		....
MT447156_|Severe_acute_respiratory_syndrome_coronavirus_2_isolate_SARS-CoV-2/human/THA/SI200383-NT/2020|_complete_genome	....		....		....
MT447157_|Severe_acute_respiratory_syndrome_coronavirus_2_isolate_SARS-CoV-2/human/THA/SI200433-NT/2020|_complete_genome	....		....		....
MT192759_|Severe_acute_respiratory_syndrome_coronavirus_2_isolate_SARS-CoV-2/human/TWN/CGMH-CGU-01/2020|_complete_genome	....		....		....
MT259229_|Severe_acute_respiratory_syndrome_coronavirus_2_isolate_SARS-CoV-2/human/CHN/Wuhan_YB012602/2020|_complete_genome	....		....		....
MT438718_|Severe_acute_respiratory_syndrome_coronavirus_2_isolate_SARS-CoV-2/human/USA/CA-CZB-1000/2020|_complete_genome	....		....		....
MT394531_|Severe_acute_respiratory_syndrome_coronavirus_2_isolate_SARS-CoV-2/human/USA/CA-CZB-59X003/2020|_complete_genome	....		....		....
MT072688_|Severe_acute_respiratory_syndrome_coronavirus_2_isolate_SARS-CoV-2/human/NPL/61-TW/2020|_complete_genome	....		....		....
MT077125_|Severe_acute_respiratory_syndrome_coronavirus_2_isolate_SARS-CoV-2/human/ITA/INMI1/2020|_complete_genome	....		....		....
LC528232_|Severe_acute_respiratory_syndrome_coronavirus_2_SARS-CoV-2/Hu/DP/Kng/19-020_RNA|_complete_genome	....		....		....
MT276597_|Severe_acute_respiratory_syndrome_coronavirus_2_isolate_SARS-CoV-2/human/ISR/ISR_JP0320/2020|_complete_genome	....		....		....
MT050493_|Severe_acute_respiratory_syndrome_coronavirus_2_isolate_SARS-CoV-2/human/IND/166/2020|_complete_genome	....		....		....
MT292572_|Severe_acute_respiratory_syndrome_coronavirus_2_isolate_SARS-CoV-2/human/ESP/Valencia11/2020|_complete_genome	....		....		....
MT256924_|Severe_acute_respiratory_syndrome_coronavirus_2_isolate_SARS-CoV-2/human/COL/79256_Antioquia/2020|_complete_genome	....		....		....
MT258377_|Severe_acute_respiratory_syndrome_coronavirus_2_isolate_SARS-CoV-2/human/USA/CZB-RR057-005/2020|_complete_genome	....		....		....
MT365032_|Severe_acute_respiratory_syndrome_coronavirus_2_isolate_SARS-CoV-2/human/HKG/HKU-904a/2020|_complete_genome	....		....		....
MT327745_|Severe_acute_respiratory_syndrome_coronavirus_2_isolate_SARS-CoV-2/human/TUR/ERAGEM-001/2020|_complete_genome	....		....		....
MT359866_|Severe_acute_respiratory_syndrome_coronavirus_2_isolate_SARS-CoV-2/human/ESP/VH198152683/2020|_complete_genome	....		....		....
MT359865_|Severe_acute_respiratory_syndrome_coronavirus_2_isolate_SARS-CoV-2/human/ESP/VH000001133/2020|_complete_genome	....		....		....
MT385436_|Severe_acute_respiratory_syndrome_coronavirus_2_isolate_SARS-CoV-2/human/USA/CA-CZB016/2020|_complete_genome	....		....		....
MT435080_|Severe_acute_respiratory_syndrome_coronavirus_2_isolate_SARS-CoV-2/human/IND/GBRC3/2020|_complete_genome	....		....		....
MT358664.1_Severe_acute_respiratory_syndrome_coronavirus_2_isolate_SARS-CoV-2/human/USA/WA-UW-4318/2020_complete_genome	....		....		....
MT451037_|Severe_acute_respiratory_syndrome_coronavirus_2_isolate_SARS-CoV-2/human/AUS/VIC131/2020|_complete_genome	....		....		....
MT300186_|Severe_acute_respiratory_syndrome_coronavirus_2_isolate_SARS-CoV-2/human/USA/UNC_200173/2020|_complete_genome	....		....		....
MT412331_|Severe_acute_respiratory_syndrome_coronavirus_2_isolate_SARS-CoV-2/human/USA/WA-UW-6399/2020|_complete_genome	....		....		....
MT418888_|Severe_acute_respiratory_syndrome_coronavirus_2_isolate_SARS-CoV-2/human/USA/VA-DCLS-0108/2020|_complete_genome	....		....		....
MT396266_|Severe_acute_respiratory_syndrome_coronavirus_2_isolate_SARS-CoV-2/mink/NLD/1/2020|_complete_genome	....		....		....
MT451038_|Severe_acute_respiratory_syndrome_coronavirus_2_isolate_SARS-CoV-2/human/AUS/VIC134/2020|_complete_genome	....		....		....
MT263074_|Severe_acute_respiratory_syndrome_coronavirus_2_isolate_SARS-CoV-2/human/PER/Peru-10/2020|_complete_genome	....		....		....
MT446312_|Severe_acute_respiratory_syndrome_coronavirus_2_isolate_SARS-CoV-2/human/Guangzhou/IQTC05/2020|_complete_genome	....		....		....
MT419812_|Severe_acute_respiratory_syndrome_coronavirus_2_isolate_SARS-CoV2/human/USA/PR-CDC-S3/2020|_complete_genome	....		....		....
MT291826_|Severe_acute_respiratory_syndrome_coronavirus_2_isolate_SARS-CoV-2/human/CHN/Wuhan_IME-WH01/2019|_complete_genome	....		....		....
MT240479_|Severe_acute_respiratory_syndrome_coronavirus_2_isolate_SARS-CoV-2/human/PAK/Gilgit1/2020|_complete_genome	....		....		....
MT320891_|Severe_acute_respiratory_syndrome_coronavirus_2_isolate_SARS-CoV-2/human/IRN/HGRC-1.1-IPI-8206/2020|_complete_genome	....		....		....
MT093571_|Severe_acute_respiratory_syndrome_coronavirus_2_isolate_SARS-CoV-2/human/SWE/01/2020|_complete_genome	....		....		....
MT039890_|Severe_acute_respiratory_syndrome_coronavirus_2_isolate_SNU01|_complete_genome	....		......................A...		....
MT007544_|Severe_acute_respiratory_syndrome_coronavirus_2_isolate_Australia/VIC01/2020|_complete_genome	....		....		....
MT291836_|Severe_acute_respiratory_syndrome_coronavirus_2_isolate_SARS-CoV-2/human/CHN/Wuhan_IME-BJ07/2020|_complete_genome	....		....		....
MT418889_|Severe_acute_respiratory_syndrome_coronavirus_2_isolate_SARS-CoV-2/human/USA/VA-DCLS-0109/2020|_complete_genome	....		....		....
MT447172_|Severe_acute_respiratory_syndrome_coronavirus_2_isolate_SARS-CoV-2/human/THA/SI205529-NT/2020|_complete_genome	....		....		....
MT215194_|Severe_acute_respiratory_syndrome_coronavirus_2_isolate_SARS-CoV-2/human/HKG.85_VM20002868/2020|_complete_genome	....		....		....
MT451882_|Severe_acute_respiratory_syndrome_coronavirus_2_isolate_SARS-CoV-2/human/IND/GBRC17b/2020|_complete_genome	....		....		....
MT428551_|Severe_acute_respiratory_syndrome_coronavirus_2_isolate_SARS-CoV-2/human/KAZ/NCB-1/2020|_complete_genome	....		....		....
MT447171_|Severe_acute_respiratory_syndrome_coronavirus_2_isolate_SARS-CoV-2/human/THA/SI205457-NST/2020|_complete_genome	....		....		....
MT451874_|Severe_acute_respiratory_syndrome_coronavirus_2_isolate_SARS-CoV-2/human/IND/GBRC10/2020|_complete_genome	....		....		....
MT412330_|Severe_acute_respiratory_syndrome_coronavirus_2_isolate_SARS-CoV-2/human/USA/CT-UW-6385/2020|_complete_genome	....		....		....
MT262993_|Severe_acute_respiratory_syndrome_coronavirus_2_isolate_SARS-Cov-2/human/PAK/Manga1/2020|_complete_genome	....		....		....

Position on the genome according to strain LC528232

B2. IP4-RdRp assay regions corresponding to the primers and the probe (73 sequences)

	15,150																		15,168		15,175																		15,193		15,237																			15,256
IP4-RdRp	G	G	T	A	A	C	T	G	G	T	A	T	G	A	T	T	T	C	G	//	T	C	A	T	A	C	A	A	A	C	C	A	C	G	C	C	A	G	G	//	C	C	T	A	T	A	T	T	A	A	C	C	T	T	G	A	C	C	A	G
NC_045512_|Severe_acute_respiratory_syndrome_coronavirus_2_isolate_Wuhan-Hu-1|_complete_genome	.	.	.	.	.	.	.	.	.	.	.	.	.	.	.	.	.	.	.		.	.	.	.	.	.	.	.	.	.	.	.	.	.	.	.	.	.	.		.	.	.	.	.	.	.	.	.	.	.	.	.	.	.	.	.	.	.	.
LC529905_|Severe_acute_respiratory_syndrome_coronavirus_2_TKYE6182_2020_RNA|_complete_genome	.	.	.	.	.	.	.	.	.	.	.	.	.	.	.	.	.	.	.		.	.	.	.	.	.	.	.	.	.	.	.	.	.	.	.	.	.	.		.	.	.	.	.	.	.	.	.	.	.	.	.	.	.	.	.	.	.	.
MT371050_|Severe_acute_respiratory_syndrome_coronavirus_2_isolate_SARS-CoV-2/human/LKA/COV486/2020|_complete_genome	.	.	.	.	.	.	.	.	.	.	.	.	.	.	.	.	.	.	.		.	.	.	.	.	.	.	.	.	.	.	.	.	.	.	.	.	.	.		.	.	.	.	.	.	.	.	.	.	.	.	.	.	.	.	.	.	.	.
LC542809_|Severe_acute_respiratory_syndrome_coronavirus_2_TKYE6947_2020_RNA|_complete_genome	.	.	.	.	.	.	.	.	.	.	.	.	.	.	.	.	.	.	.		.	.	.	.	.	.	.	.	.	.	.	.	.	.	.	.	.	.	.		.	.	.	.	.	.	.	.	.	.	.	.	.	.	.	.	.	.	.	.
MT135042_|Severe_acute_respiratory_syndrome_coronavirus_2_isolate_SARS-CoV-2/human/CHN/231/2020|_complete_genome	.	.	.	.	.	.	.	.	.	.	.	.	.	.	.	.	.	.	.		.	.	.	.	.	.	.	.	.	.	.	.	.	.	.	.	.	.	.		.	.	.	.	.	.	.	.	.	.	.	.	.	.	.	.	.	.	.	.
MT350282_|Severe_acute_respiratory_syndrome_coronavirus_2_isolate_SARS-CoV-2/human/BRA/SP02cc/2020|_complete_genome	.	.	.	.	.	.	.	.	.	.	.	.	.	.	.	.	.	.	.		.	.	.	.	.	.	.	.	.	.	.	.	.	.	.	.	.	.	.		.	.	.	.	.	.	.	.	.	.	.	.	.	.	.	.	.	.	.	.
MT114412_|Severe_acute_respiratory_syndrome_coronavirus_2_isolate_SARS-CoV-2/human/HKG/HKU-902a/2020|_complete_genome	.	.	.	.	.	.	.	.	.	.	.	.	.	.	.	.	.	.	.		.	.	.	.	.	.	.	.	.	.	.	.	.	.	.	.	.	.	.		.	.	.	.	.	.	.	.	.	.	.	.	.	.	.	.	.	.	.	.
MT192772_|Severe_acute_respiratory_syndrome_coronavirus_2_isolate_SARS-CoV-2/human/VNM/nCoV-19-01S/2020|_complete_genome	.	.	.	.	.	.	.	.	.	.	.	.	.	.	.	.	.	.	.		.	.	.	.	.	.	.	.	.	.	.	.	.	.	.	.	.	.	.		.	.	.	.	.	.	.	.	.	.	.	.	.	.	.	.	.	.	.	.
MT192773_|Severe_acute_respiratory_syndrome_coronavirus_2_isolate_SARS-CoV-2/human/VNM/nCoV-19-02S/2020|_complete_genome	.	.	.	.	.	.	.	.	.	.	.	.	.	.	.	.	.	.	.		.	.	.	.	.	.	.	.	.	.	.	.	.	.	.	.	.	.	.		.	.	.	.	.	.	.	.	.	.	.	.	.	.	.	.	.	.	.	.
MT320538_|Severe_acute_respiratory_syndrome_coronavirus_2_isolate_SARS-CoV-2/human/FRA/KRA-ROB/2020|_complete_genome	.	.	.	.	.	.	.	.	.	.	.	.	.	.	.	.	.	.	.		.	.	.	.	.	.	.	.	.	.	.	.	.	.	.	.	.	.	.		.	.	.	.	.	.	.	.	.	.	.	.	.	.	.	.	.	.	.	.
MT276330_|Severe_acute_respiratory_syndrome_coronavirus_2_isolate_SARS-CoV-2/human/USA/FL_5091/2020|_complete_genome	.	.	.	.	.	.	.	.	.	.	.	.	.	.	.	.	.	.	.		.	.	.	.	.	.	.	.	.	.	.	.	.	.	.	.	.	.	.		.	.	.	.	.	.	.	.	.	.	.	.	.	.	.	.	.	.	.	.
MT304491_|Severe_acute_respiratory_syndrome_coronavirus_2_isolate_SARS-CoV-2/human/USA/TX_2967/2020|_complete_genome	.	.	.	.	.	.	.	.	.	.	.	.	.	.	.	.	.	.	.		.	.	.	.	.	.	.	.	.	.	.	.	.	.	.	.	.	.	.		.	.	.	.	.	.	.	.	.	.	.	.	.	.	.	.	.	.	.	.
MT159722_|Severe_acute_respiratory_syndrome_coronavirus_2_isolate_2019-nCoV/USA-CruiseA-6/2020|_complete_genome	.	.	.	.	.	.	.	.	.	.	.	.	.	.	.	.	.	.	.		.	.	.	.	.	.	.	.	.	.	.	.	.	.	.	.	.	.	.		.	.	.	.	.	.	.	.	.	.	.	.	.	.	.	.	.	.	.	.
MT452574_|Severe_acute_respiratory_syndrome_coronavirus_2_isolate_SAR-CoV-2/human/USA/NY-QDX-00000001/2020|_complete_genome	.	.	.	.	.	.	.	.	.	.	.	.	.	.	.	.	.	.	.		.	.	.	.	.	.	.	.	.	.	.	.	.	.	.	.	.	.	.		.	.	.	.	.	.	.	.	.	.	.	.	.	.	.	.	.	.	.	.
MT422807_|Severe_acute_respiratory_syndrome_coronavirus_2_isolate_SARS-CoV-2/human/USA/UF-5/2020|_complete_genome	.	.	.	.	.	.	.	.	.	.	.	.	.	.	.	.	.	.	.		.	.	.	.	.	.	.	.	.	.	.	.	.	.	.	.	.	.	.		.	.	.	.	.	.	.	.	.	.	.	.	.	.	.	.	.	.	.	.
MT339041_|Severe_acute_respiratory_syndrome_coronavirus_2_isolate_SARS-CoV-2/human/USA/AZ-ASU2936/2020|_complete_genome	.	.	.	.	.	.	.	.	.	.	.	.	.	.	.	.	.	.	.		.	.	.	.	.	.	.	.	.	.	.	.	.	.	.	.	.	.	.		.	.	.	.	.	.	.	.	.	.	.	.	.	.	.	.	.	.	.	.
MT328035_|Severe_acute_respiratory_syndrome_coronavirus_2_isolate_SARS-CoV-2/human/GRC/13/2020|_complete_genome	.	.	.	.	.	.	.	.	.	.	.	.	.	.	.	.	.	.	.		.	.	.	.	.	.	.	.	.	.	.	.	.	.	.	.	.	.	.		.	.	.	.	.	.	.	.	.	.	.	.	.	.	.	.	.	.	.	.
MT121215_|Severe_acute_respiratory_syndrome_coronavirus_2_isolate_SARS-CoV-2/human/CHN/SH01/2020|_complete_genome	.	.	.	.	.	.	.	.	.	.	.	.	.	.	.	.	.	.	.		.	.	.	.	.	.	.	.	.	.	.	.	.	.	.	.	.	.	.		.	.	.	.	.	.	.	.	.	.	.	.	.	.	.	.	.	.	.	.
MT039874_|Severe_acute_respiratory_syndrome_coronavirus_2_isolate_SARS-CoV-2/human/CHN/IME-HZ01/2020|_complete_genome	.	.	.	.	.	.	.	.	.	.	.	.	.	.	.	.	.	.	.		.	.	.	.	.	.	.	.	.	.	.	.	.	.	.	.	.	.	.		.	.	.	.	.	.	.	.	.	.	.	.	.	.	.	.	.	.	.	.
MT066175_|Severe_acute_respiratory_syndrome_coronavirus_2_isolate_SARS-CoV-2/NTU01/TWN/human/2020|_complete_genome	.	.	.	.	.	.	.	.	.	.	.	.	.	.	.	.	.	.	.		.	.	.	.	.	.	.	.	.	.	.	.	.	.	.	.	.	.	.		.	.	.	.	.	.	.	.	.	.	.	.	.	.	.	.	.	.	.	.
MT020781_|Severe_acute_respiratory_syndrome_coronavirus_2_isolate_nCoV-FIN-29-Jan-2020|_partial_genome	.	.	.	.	.	.	.	.	.	.	.	.	.	.	.	.	.	.	.		.	.	.	.	.	.	.	.	.	.	.	.	.	.	.	.	.	.	.		.	.	.	.	.	.	.	.	.	.	.	.	.	.	.	.	.	.	.	.
MT066156_|Severe_acute_respiratory_syndrome_coronavirus_2_isolate_SARS-CoV-2/human/ITA/INMI1/2020|_complete_genome	.	.	.	.	.	.	.	.	.	.	.	.	.	.	.	.	.	.	.		.	.	.	.	.	.	.	.	.	.	.	.	.	.	.	.	.	.	.		.	.	.	.	.	.	.	.	.	.	.	.	.	.	.	.	.	.	.	.
MT281577_|Severe_acute_respiratory_syndrome_coronavirus_2_isolate_SARS-CoV-2/human/CHN/Fuyang_FY002/2020|_complete_genome	.	.	.	.	.	.	.	.	.	.	.	.	.	.	.	.	.	.	.		.	.	.	.	.	.	.	.	.	.	.	.	.	.	.	.	.	.	.		.	.	.	.	.	.	.	.	.	.	.	.	.	.	.	.	.	.	.	.
MT304474_|Severe_acute_respiratory_syndrome_coronavirus_2_isolate_SARS-CoV-2/human/KOR/BA-ACH_2604/2020|_complete_genome	.	.	.	.	.	.	.	.	.	.	.	.	.	.	.	.	.	.	.		.	.	.	.	.	.	.	.	.	.	.	.	.	.	.	.	.	.	.		.	.	.	.	.	.	.	.	.	.	.	.	.	.	.	.	.	.	.	.
MT396241_|Severe_acute_respiratory_syndrome_coronavirus_2_isolate_SARS-CoV-2/human/CHN/YN-0306-466/2020|_complete_genome	.	.	.	.	.	.	.	.	.	.	.	.	.	.	.	.	.	.	.		.	.	.	.	.	.	.	.	.	.	.	.	.	.	.	.	.	.	.		.	.	.	.	.	.	.	.	.	.	.	.	.	.	.	.	.	.	.	.
MT434782_|Severe_acute_respiratory_syndrome_coronavirus_2_isolate_SARS-CoV-2/human/USA/NY-CDC-SURV0076NYC/2020|_complete_genome	.	.	.	.	.	.	.	.	.	.	.	.	.	.	.	.	.	.	.		.	.	.	.	.	.	.	.	.	.	.	.	.	.	.	.	.	.	.		.	.	.	.	.	.	.	.	.	.	.	.	.	.	.	.	.	.	.	.
MT106054_|Severe_acute_respiratory_syndrome_coronavirus_2_isolate_2019-nCoV/USA-TX1/2020|_complete_genome	.	.	.	.	.	.	.	.	.	.	.	.	.	.	.	.	.	.	.		.	.	.	.	.	.	.	.	.	.	.	.	.	.	.	.	.	.	.		.	.	.	.	.	.	.	.	.	.	.	.	.	.	.	.	.	.	.	.
MT447156_|Severe_acute_respiratory_syndrome_coronavirus_2_isolate_SARS-CoV-2/human/THA/SI200383-NT/2020|_complete_genome	.	.	.	.	.	.	.	.	.	.	.	.	.	.	.	.	.	.	.		.	.	.	.	.	.	.	.	.	.	.	.	.	.	.	.	.	.	.		.	.	.	.	.	.	.	.	.	.	.	.	.	.	.	.	.	.	.	.
MT447157_|Severe_acute_respiratory_syndrome_coronavirus_2_isolate_SARS-CoV-2/human/THA/SI200433-NT/2020|_complete_genome	.	.	.	.	.	.	.	.	.	.	.	.	.	.	.	.	.	.	.		.	.	.	.	.	.	.	.	.	.	.	.	.	.	.	.	.	.	.		.	.	.	.	.	.	.	.	.	.	.	.	.	.	.	.	.	.	.	.
MT192759_|Severe_acute_respiratory_syndrome_coronavirus_2_isolate_SARS-CoV-2/human/TWN/CGMH-CGU-01/2020|_complete_genome	.	.	.	.	.	.	.	.	.	.	.	.	.	.	.	.	.	.	.		.	.	.	.	.	.	.	.	.	.	.	.	.	.	.	.	.	.	.		.	.	.	.	.	.	.	.	.	.	.	.	.	.	.	.	.	.	.	.
MT259229_|Severe_acute_respiratory_syndrome_coronavirus_2_isolate_SARS-CoV-2/human/CHN/Wuhan_YB012602/2020|_complete_genome	.	.	.	.	.	.	.	.	.	.	.	.	.	.	.	.	.	.	.		.	.	.	.	.	.	.	.	.	.	.	.	.	.	.	.	.	.	.		.	.	.	.	.	.	.	.	.	.	.	.	.	.	.	.	.	.	.	.
MT438718_|Severe_acute_respiratory_syndrome_coronavirus_2_isolate_SARS-CoV-2/human/USA/CA-CZB-1000/2020|_complete_genome	.	.	.	.	.	.	.	.	.	.	.	.	.	.	.	.	.	.	.		.	.	.	.	.	.	.	.	.	.	.	.	.	.	.	.	.	.	.		.	.	.	.	.	.	.	.	.	.	.	.	.	.	.	.	.	.	.	.
MT394531_|Severe_acute_respiratory_syndrome_coronavirus_2_isolate_SARS-CoV-2/human/USA/CA-CZB-59X003/2020|_complete_genome	.	.	.	.	.	.	.	.	.	.	.	.	.	.	.	.	.	.	.		.	.	.	.	.	.	.	.	.	.	.	.	.	.	.	.	.	.	.		.	.	.	.	.	.	.	.	.	.	.	.	.	.	.	.	.	.	.	.
MT072688_|Severe_acute_respiratory_syndrome_coronavirus_2_isolate_SARS-CoV-2/human/NPL/61-TW/2020|_complete_genome	.	.	.	.	.	.	.	.	.	.	.	.	.	.	.	.	.	.	.		.	.	.	.	.	.	.	.	.	.	.	.	.	.	.	.	.	.	.		.	.	.	.	.	.	.	.	.	.	.	.	.	.	.	.	.	.	.	.
MT077125_|Severe_acute_respiratory_syndrome_coronavirus_2_isolate_SARS-CoV-2/human/ITA/INMI1/2020|_complete_genome	.	.	.	.	.	.	.	.	.	.	.	.	.	.	.	.	.	.	.		.	.	.	.	.	.	.	.	.	.	.	.	.	.	.	.	.	.	.		.	.	.	.	.	.	.	.	.	.	.	.	.	.	.	.	.	.	.	.
LC528232_|Severe_acute_respiratory_syndrome_coronavirus_2_SARS-CoV-2/Hu/DP/Kng/19-020_RNA|_complete_genome	.	.	.	.	.	.	.	.	.	.	.	.	.	.	.	.	.	.	.		.	.	.	.	.	.	.	.	.	.	.	.	.	.	.	.	.	.	.		.	.	.	.	.	.	.	.	.	.	.	.	.	.	.	.	.	.	.	.
MT276597_|Severe_acute_respiratory_syndrome_coronavirus_2_isolate_SARS-CoV-2/human/ISR/ISR_JP0320/2020|_complete_genome	.	.	.	.	.	.	.	.	.	.	.	.	.	.	.	.	.	.	.		.	.	.	.	.	.	.	.	.	.	.	.	.	.	.	.	.	.	.		.	.	.	.	.	.	.	.	.	.	.	.	.	.	.	.	.	.	.	.
MT050493_|Severe_acute_respiratory_syndrome_coronavirus_2_isolate_SARS-CoV-2/human/IND/166/2020|_complete_genome	.	.	.	.	.	.	.	.	.	.	.	.	.	.	.	.	.	.	.		.	.	.	.	.	.	.	.	.	.	.	.	.	.	.	.	.	.	.		.	.	.	.	.	.	.	.	.	.	.	.	.	.	.	.	.	.	.	.
MT292572_|Severe_acute_respiratory_syndrome_coronavirus_2_isolate_SARS-CoV-2/human/ESP/Valencia11/2020|_complete_genome	.	.	.	.	.	.	.	.	.	.	.	.	.	.	.	.	.	.	.		.	.	.	.	.	.	.	.	.	.	.	.	.	.	.	.	.	.	.		.	.	.	.	.	.	.	.	.	.	.	.	.	.	.	.	.	.	.	.
MT256924_|Severe_acute_respiratory_syndrome_coronavirus_2_isolate_SARS-CoV-2/human/COL/79256_Antioquia/2020|_complete_genome	.	.	.	.	.	.	.	.	.	.	.	.	.	.	.	.	.	.	.		.	.	.	.	.	.	.	.	.	.	.	.	.	.	.	.	.	.	.		.	.	.	.	.	.	.	.	.	.	.	.	.	.	.	.	.	.	.	.
MT258377_|Severe_acute_respiratory_syndrome_coronavirus_2_isolate_SARS-CoV-2/human/USA/CZB-RR057-005/2020|_complete_genome	.	.	.	.	.	.	.	.	.	.	.	.	.	.	.	.	.	.	.		.	.	.	.	.	.	.	.	.	.	.	.	.	.	.	.	.	.	.		.	.	.	.	.	.	.	.	.	.	.	.	.	.	.	.	.	.	.	.
MT365032_|Severe_acute_respiratory_syndrome_coronavirus_2_isolate_SARS-CoV-2/human/HKG/HKU-904a/2020|_complete_genome	.	.	.	.	.	.	.	.	.	.	.	.	.	.	.	.	.	.	.		.	.	.	.	.	.	.	.	.	.	.	.	.	.	.	.	.	.	.		.	.	.	.	.	.	.	.	.	.	.	.	.	.	.	.	.	.	.	.
MT327745_|Severe_acute_respiratory_syndrome_coronavirus_2_isolate_SARS-CoV-2/human/TUR/ERAGEM-001/2020|_complete_genome	.	.	.	.	.	.	.	.	.	.	.	.	.	.	.	.	.	.	.		.	.	.	.	.	.	.	.	.	.	.	.	.	.	.	.	.	.	.		.	.	.	.	.	.	.	.	.	.	.	.	.	.	.	.	.	.	.	.
MT359866_|Severe_acute_respiratory_syndrome_coronavirus_2_isolate_SARS-CoV-2/human/ESP/VH198152683/2020|_complete_genome	.	.	.	.	.	.	.	.	.	.	.	.	.	.	.	.	.	.	.		.	.	.	.	.	.	.	.	.	.	.	.	.	.	.	.	.	.	.		.	.	.	.	.	.	.	.	.	.	.	.	.	.	.	.	.	.	.	.
MT359865_|Severe_acute_respiratory_syndrome_coronavirus_2_isolate_SARS-CoV-2/human/ESP/VH000001133/2020|_complete_genome	.	.	.	.	.	.	.	.	.	.	.	.	.	.	.	.	.	.	.		.	.	.	.	.	.	.	.	.	.	.	.	.	.	.	.	.	.	.		.	.	.	.	.	.	.	.	.	.	.	.	.	.	.	.	.	.	.	.
MT385436_|Severe_acute_respiratory_syndrome_coronavirus_2_isolate_SARS-CoV-2/human/USA/CA-CZB016/2020|_complete_genome	.	.	.	.	.	.	.	.	.	.	.	.	.	.	.	.	.	.	.		.	.	.	.	.	.	.	.	.	.	.	.	.	.	.	.	.	.	.		.	.	.	.	.	.	.	.	.	.	.	.	.	.	.	.	.	.	.	.
MT435080_|Severe_acute_respiratory_syndrome_coronavirus_2_isolate_SARS-CoV-2/human/IND/GBRC3/2020|_complete_genome	.	.	.	.	.	.	.	.	.	.	.	.	.	.	.	.	.	.	.		.	.	.	.	.	.	.	.	.	.	.	.	.	.	.	.	.	.	.		.	.	.	.	.	.	.	.	.	.	.	.	.	.	.	.	.	.	.	.
MT358664.1_Severe_acute_respiratory_syndrome_coronavirus_2_isolate_SARS-CoV-2/human/USA/WA-UW-4318/2020_complete_genome	.	.	.	.	.	.	.	.	.	.	.	.	.	.	.	.	.	.	.		.	.	.	.	.	.	.	.	.	.	.	.	.	.	.	.	.	.	.		.	.	.	.	.	.	.	.	.	.	.	.	.	.	.	.	.	.	.	.
MT451037_|Severe_acute_respiratory_syndrome_coronavirus_2_isolate_SARS-CoV-2/human/AUS/VIC131/2020|_complete_genome	.	.	.	.	.	.	.	.	.	.	.	.	.	.	.	.	.	.	.		.	.	.	.	.	.	.	.	.	.	.	.	.	.	.	.	.	.	.		.	.	.	.	.	.	.	.	.	.	.	.	.	.	.	.	.	.	.	.
MT300186_|Severe_acute_respiratory_syndrome_coronavirus_2_isolate_SARS-CoV-2/human/USA/UNC_200173/2020|_complete_genome	.	.	.	.	.	.	.	.	.	.	.	.	.	.	.	.	.	.	.		.	.	.	.	.	.	.	.	.	.	.	.	.	.	.	.	.	.	.		.	.	.	.	.	.	.	.	.	.	.	.	.	.	.	.	.	.	.	.
MT412331_|Severe_acute_respiratory_syndrome_coronavirus_2_isolate_SARS-CoV-2/human/USA/WA-UW-6399/2020|_complete_genome	.	.	.	.	.	.	.	.	.	.	.	.	.	.	.	.	.	.	.		.	.	.	.	.	.	.	.	.	.	.	.	.	.	.	.	.	.	.		.	.	.	.	.	.	.	.	.	.	.	.	.	.	.	.	.	.	.	.
MT418888_|Severe_acute_respiratory_syndrome_coronavirus_2_isolate_SARS-CoV-2/human/USA/VA-DCLS-0108/2020|_complete_genome	.	.	.	.	.	.	.	.	.	.	.	.	.	.	.	.	.	.	.		.	.	.	.	.	.	.	.	.	.	.	.	.	.	.	.	.	.	.		.	.	.	.	.	.	.	.	.	.	.	.	.	.	.	.	.	.	.	.
MT396266_|Severe_acute_respiratory_syndrome_coronavirus_2_isolate_SARS-CoV-2/mink/NLD/1/2020|_complete_genome	.	.	.	.	.	.	.	.	.	.	.	.	.	.	.	.	.	.	.		.	.	.	.	.	.	.	.	.	.	.	.	.	.	.	.	.	.	.		.	.	.	.	.	.	.	.	.	.	.	.	.	.	.	.	.	.	.	.
MT451038_|Severe_acute_respiratory_syndrome_coronavirus_2_isolate_SARS-CoV-2/human/AUS/VIC134/2020|_complete_genome	.	.	.	.	.	.	.	.	.	.	.	.	.	.	.	.	.	.	.		.	.	.	.	.	.	.	.	.	.	.	.	.	.	.	.	.	.	.		.	.	.	.	.	.	.	.	.	.	.	.	.	.	.	.	.	.	.	.
MT263074_|Severe_acute_respiratory_syndrome_coronavirus_2_isolate_SARS-CoV-2/human/PER/Peru-10/2020|_complete_genome	.	.	.	.	.	.	.	.	.	.	.	.	.	.	.	.	.	.	.		.	.	.	.	.	.	.	.	.	.	.	.	.	.	.	.	.	.	.		.	.	.	.	.	.	.	.	.	.	.	.	.	.	.	.	.	.	.	.
MT446312_|Severe_acute_respiratory_syndrome_coronavirus_2_isolate_SARS-CoV-2/human/Guangzhou/IQTC05/2020|_complete_genome	.	.	.	.	.	.	.	.	.	.	.	.	.	.	.	.	.	.	.		.	.	.	.	.	.	.	.	.	.	.	.	.	.	.	.	.	.	.		.	.	.	.	.	.	.	.	.	.	.	.	.	.	.	.	.	.	.	.
MT419812_|Severe_acute_respiratory_syndrome_coronavirus_2_isolate_SARS-CoV2/human/USA/PR-CDC-S3/2020|_complete_genome	.	.	.	.	.	.	.	.	.	.	.	.	.	.	.	.	.	.	.		.	.	.	.	.	.	.	.	.	.	.	.	.	.	.	.	.	.	.		.	.	.	.	.	.	.	.	.	.	.	.	.	.	.	.	.	.	.	.
MT291826_|Severe_acute_respiratory_syndrome_coronavirus_2_isolate_SARS-CoV-2/human/CHN/Wuhan_IME-WH01/2019|_complete_genome	.	.	.	.	.	.	.	.	.	.	.	.	.	.	.	.	.	.	.		.	.	.	.	.	.	.	.	.	.	.	.	.	.	.	.	.	.	.		.	.	.	.	.	.	.	.	.	.	.	.	.	.	.	.	.	.	.	.
MT240479_|Severe_acute_respiratory_syndrome_coronavirus_2_isolate_SARS-CoV-2/human/PAK/Gilgit1/2020|_complete_genome	.	.	.	.	.	.	.	.	.	.	.	.	.	.	.	.	.	.	.		.	.	.	.	.	.	.	.	.	.	.	.	.	.	.	.	.	.	.		.	.	.	.	.	.	.	.	.	.	.	.	.	.	.	.	.	.	.	.
MT320891_|Severe_acute_respiratory_syndrome_coronavirus_2_isolate_SARS-CoV-2/human/IRN/HGRC-1.1-IPI-8206/2020|_complete_genome	.	.	.	.	.	.	.	.	.	.	.	.	.	.	.	.	.	.	.		.	.	.	.	.	.	.	.	.	.	.	.	.	.	.	.	.	.	.		.	.	.	.	.	.	.	.	.	.	.	.	.	.	.	.	.	.	.	.
MT093571_|Severe_acute_respiratory_syndrome_coronavirus_2_isolate_SARS-CoV-2/human/SWE/01/2020|_complete_genome	.	.	.	.	.	.	.	.	.	.	.	.	.	.	.	.	.	.	.		.	.	.	.	.	.	.	.	.	.	.	.	.	.	.	.	.	.	.		.	.	.	.	.	.	.	.	.	.	.	.	.	.	.	.	.	.	.	.
MT039890_|Severe_acute_respiratory_syndrome_coronavirus_2_isolate_SNU01|_complete_genome	.	.	.	.	.	.	.	.	.	.	.	.	.	.	.	.	.	.	.		.	.	.	.	.	.	.	.	.	.	.	.	.	.	.	.	.	.	.		.	.	.	.	.	.	.	.	.	.	.	.	.	.	.	.	.	.	.	.
MT007544_|Severe_acute_respiratory_syndrome_coronavirus_2_isolate_Australia/VIC01/2020|_complete_genome	.	.	.	.	.	.	.	.	.	.	.	.	.	.	.	.	.	.	.		.	.	.	.	.	.	.	.	.	.	.	.	.	.	.	.	.	.	.		.	.	.	.	.	.	.	.	.	.	.	.	.	.	.	.	.	.	.	.
MT291836_|Severe_acute_respiratory_syndrome_coronavirus_2_isolate_SARS-CoV-2/human/CHN/Wuhan_IME-BJ07/2020|_complete_genome	.	.	.	.	.	.	.	.	.	.	.	.	.	.	.	.	.	.	.		.	.	.	.	.	.	.	.	.	.	.	.	.	.	.	.	.	.	.		.	.	.	.	.	.	.	.	.	.	.	.	.	.	.	.	.	.	.	.
MT418889_|Severe_acute_respiratory_syndrome_coronavirus_2_isolate_SARS-CoV-2/human/USA/VA-DCLS-0109/2020|_complete_genome	.	.	.	.	.	.	.	.	.	.	.	.	.	.	.	.	.	.	.		.	.	.	.	.	.	.	.	.	.	.	.	.	.	.	.	.	.	.		.	.	.	.	.	.	.	.	.	.	.	.	.	.	.	.	.	.	.	.
MT447172_|Severe_acute_respiratory_syndrome_coronavirus_2_isolate_SARS-CoV-2/human/THA/SI205529-NT/2020|_complete_genome	.	.	.	.	.	.	.	.	.	.	.	.	.	.	.	.	.	.	.		.	.	.	.	.	.	.	.	.	.	.	.	.	.	.	.	.	.	.		.	.	.	.	.	.	.	.	.	.	.	.	.	.	.	.	.	.	.	.
MT215194_|Severe_acute_respiratory_syndrome_coronavirus_2_isolate_SARS-CoV-2/human/HKG.85_VM20002868/2020|_complete_genome	.	.	.	.	.	.	.	.	.	.	.	.	.	.	.	.	.	.	.		.	.	.	.	.	.	.	.	.	.	.	.	.	.	.	.	.	.	.		.	.	.	.	.	.	.	.	.	.	.	.	.	.	.	.	.	.	.	.
MT451882_|Severe_acute_respiratory_syndrome_coronavirus_2_isolate_SARS-CoV-2/human/IND/GBRC17b/2020|_complete_genome	.	.	.	.	.	.	.	.	.	.	.	.	.	.	.	.	.	.	.		.	.	.	.	.	.	.	.	.	.	.	.	.	.	.	.	.	.	.		.	.	.	.	.	.	.	.	.	.	.	.	.	.	.	.	.	.	.	.
MT428551_|Severe_acute_respiratory_syndrome_coronavirus_2_isolate_SARS-CoV-2/human/KAZ/NCB-1/2020|_complete_genome	.	.	.	.	.	.	.	.	.	.	.	.	.	.	.	.	.	.	.		.	.	.	.	.	.	.	.	.	.	.	.	.	.	.	.	.	.	.		.	.	.	.	.	.	.	.	.	.	.	.	.	.	.	.	.	.	.	.
MT447171_|Severe_acute_respiratory_syndrome_coronavirus_2_isolate_SARS-CoV-2/human/THA/SI205457-NST/2020|_complete_genome	.	.	.	.	.	.	.	.	.	.	.	.	.	.	.	.	.	.	.		.	.	.	.	.	.	.	.	.	.	.	.	.	.	.	.	.	.	.		.	.	.	.	.	.	.	.	.	.	.	.	.	.	.	.	.	.	.	.
MT451874_|Severe_acute_respiratory_syndrome_coronavirus_2_isolate_SARS-CoV-2/human/IND/GBRC10/2020|_complete_genome	.	.	.	.	.	.	.	.	.	.	.	.	.	.	.	.	.	.	.		.	.	.	.	.	.	.	.	.	.	.	.	.	.	.	.	.	.	.		.	.	.	.	.	.	.	.	.	.	.	.	.	.	.	.	.	.	.	.
MT412330_|Severe_acute_respiratory_syndrome_coronavirus_2_isolate_SARS-CoV-2/human/USA/CT-UW-6385/2020|_complete_genome	.	.	.	.	.	.	.	.	.	.	.	.	.	.	.	.	.	.	.		.	.	.	.	.	.	.	.	.	.	.	.	.	.	.	.	.	.	.		.	.	.	.	.	.	.	.	.	.	.	.	.	.	.	.	.	.	.	.
MT262993_|Severe_acute_respiratory_syndrome_coronavirus_2_isolate_SARS-Cov-2/human/PAK/Manga1/2020|_complete_genome	.	.	.	.	.	.	.	.	.	.	.	.	.	.	.	.	.	.	.		.	.	.	.	.	.	.	.	.	.	.	.	.	.	.	.	.	.	.		.	.	.	.	.	.	.	.	.	.	.	.	.	.	.	.	.	.	.	.

Position on the genome according to strain LC528232
